# Time affluence and health: a scoping review of conceptual and methodological patterns

**DOI:** 10.3389/fpubh.2026.1824268

**Published:** 2026-07-15

**Authors:** Sang Yong Yoon

**Affiliations:** Taekwondo Studies at the Lifelong Education Institute, Dankook University, Yongin, Republic of Korea

**Keywords:** health, life satisfaction, scoping review, time affluence, wellbeing

## Abstract

This scoping review systematically maps how the conceptualization of time affluence, its measurement, and its links to health and wellbeing across existing literature. Drawing on conceptual and theoretical papers, direct empirical investigations, and proxy-based empirical studies, this review identifies substantial variations in conceptual definitions and measurement approaches. The evidence generally frames time affluence as a subjective psychological resource that is associated with psychosocial wellbeing, although structurally oriented studies examine temporal availability using proxy indicators. Across study types, research has primarily focused on psychological outcomes, paying only limited attention to behavioral, physiological, and population-level indicators of health. This review highlights the need for greater conceptual integration between subjective and structural perspectives, methodological diversification going beyond cross-sectional self-report designs, and broader public health investigation of temporal resources as potential determinants for health equity.

## 1 Introduction

Time is a universal but unequally distributed resource. In particular, the structuring of working hours following industrialization and the acceleration of life have contributed to an increasingly compressed experience of time, which has led many to perceive that they are constantly pressed for time (1). Such subjective experiences of time pressure or the scarcity of time extend beyond the minor inconveniences of daily life and are closely associated with stress, poorer mental health outcomes, and reduced life satisfaction (2). In addition, growing evidence suggests that time scarcity could function as a constraint on health-related behaviors, including participation in physical activities, positioning temporal conditions as an important social determinant of both mental and physical health (3).

In contrast to this deficit-oriented perspective, time affluence captures a more positive and resource-based temporal experience. Time affluence was explicitly conceptualized by Kasser and Sheldon (4), who defined it as a subjective perception of having sufficient time and autonomy over the use of one's time rather than as any objective amount of free time. This concept emphasizes perceived control, flexibility, and freedom in time use, which distinguishes it from purely quantitative indicators such as working hours or duration of leisure time. Empirical research also shows that higher levels of perceived time affluence are associated with greater psychological wellbeing, lower stress, and higher life satisfaction (4, 5). Time affluence thus serves as an important counter-concept to time scarcity and provides a useful framework for the understanding of how subjective temporal experiences can enable health-promoting behaviors and enhance the overall quality of life.

The study of health and wellbeing has predominantly examined temporal conditions through such concepts as time scarcity, time pressure, and time poverty [e.g., (1, 6, 7)]. This research has consistently demonstrated that perceived time shortages are associated with elevated stress levels, lower subjective wellbeing and life satisfaction, and greater difficulty in performing physical activity and other health-promoting behaviors. Thus, time has largely been conceptualized as a constraining or risk factor undermining health-related behaviors and psychological wellbeing (6). However, the experience of time cannot be adequately explained solely in terms of whether free time is actually scarce or abundant (1). Even under similar objective temporal conditions, some may perceive their time as sufficient or, conversely, may feel persistently pressed for time, and this subjective perception is closely linked to quality of life. Against this backdrop, a growing body of research has begun to focus on time affluence as a conceptual counterpoint to time scarcity (8). Time affluence refers to the subjective experience of having sufficient time along with a perceived sense of control over how that time can be allocated and to meaningful activities (4).

In social psychology, time affluence has been conceptualized not merely as the amount of leisure time available but also as a psychological resource. Earlier work on subjective time experience has found that individuals' perceptions of time sufficiency and temporal control are closely linked to autonomy and wellbeing (9, 10). Building on this perspective, Kasser and Sheldon (4) conceptualized time affluence as an immaterial resource that is distinct from material affluence, finding empirically that it contributes to life satisfaction and positive affect, independently of income level. In addition, Rudd (3) situated time scarcity and time affluence along a single conceptual continuum, which suggests that greater perceived sufficiency of time is associated with enhanced emotional stability and overall wellbeing. In terms of research into political economy and wellbeing, efforts have also been made to conceptualize time affluence as a more structural condition. Burchardt and Ickler (5) defined time affluence as the availability of time enabling individuals to engage in meaningful and relational activities, considering it a key dimension that complements income-centered indicators of wellbeing. This perspective aligns with broader arguments that time constitutes a critical yet unevenly distributed resource that shapes quality of life and wellbeing (11).

Despite interest that is growing across disciplines, the literature on time affluence and health remains characterized by substantial conceptual heterogeneity. More broadly, the study of time and wellbeing has long identified tensions between objective temporal indicators and subjective experiences of time, with time availability, time pressure, and perceived time sufficiency often being treated interchangeably although they represent distinct constructs (10, 12). Here, some studies define time affluence simply as the inverse of time scarcity, where others adopt broader conceptualizations that emphasize autonomy, relational engagement, and participation in meaningful activities (5). Thus, time affluence has been embedded in divergent theoretical frameworks, and consensus on its core components remains limited.

Second, there is a marked imbalance in terms measurement approaches. Empirical studies directly assessing time affluence remain relatively scarce, and among those that do exist, many rely on the Multi-dimensional Time Affluence Scale (MATAS) developed by Kasser and Sheldon (4). While these studies consistently identify positive associations between perceived time affluence and psychological wellbeing, life satisfaction, and reduced stress, most use cross-sectional designs, thereby limiting their ability to make causal inferences (4, 13). More generally, scholars have cautioned that reliance on single-time-point assessments may not adequately capture the dynamic and experiential nature of time-related resources (2).

However, a substantial body of research has approached time affluence indirectly, employing proxy indicators such as discretionary time, working hours, or time-saving behaviors. Studies that adopt this approach have documented non-linear associations between discretionary time and wellbeing, which suggests that insufficient and excessive time may be linked to lower life satisfaction (8, 14). However, prior research has also emphasized that discretionary time and similar objective indicators may fail to reflect how time is subjectively experienced and valued in everyday life, rendering such proxies conceptually distant from time affluence as a psychological construct (2, 15). Third, the literature is fragmented in terms of research approaches. While conceptual and theoretical papers, direct empirical studies, and proxy-based empirical studies each offer valuable insights into the relationship between time and health, relatively little effort has been expended on systematically comparing and synthesizing these bodies of work in a single analytical framework. Moreover, health-related outcomes as examined across studies are highly dispersed, including subjective wellbeing, life satisfaction, stress, mindfulness, and related indicators. This dispersion hinders the development of an integrated understanding of the ways in which time affluence is linked to health.

Thus, this study took a scoping review approach, as this is more appropriate than a systematic review or meta-analysis focused on effect sizes or causal relationships for the state of the field. Scoping reviews are designed to explore how a given concept is defined, operationalized, and examined across diverse bodies of literature while also identifying conceptual heterogeneity and research gaps. Thus, categorizing existing studies on time affluence and health into three groups, namely, conceptual and theoretical papers, direct empirical studies, and proxy-based empirical studies, can provide a structured approach to the advancement of a more comprehensive understanding of this emerging research field. The purpose of this study is to systematically examine the literature on the relationship between time affluence and health and to structure the ways that the concept has been conceptualized and empirically investigated. Therefore, the following research questions were formulated:

**Research Question 1 (RQ1):** How is time affluence conceptualized and framed in research across disciplines?
**Research Question 2 (RQ2):** What research designs and measurement approaches are used to examine the relationship between time affluence and health or wellbeing?
**Research Question 3 (RQ3):** How is time affluence associated with health and wellbeing outcomes in the literature, and what common patterns and limitations can be identified?

Through addressing these research questions, this scoping review is intended to provide an integrative overview of how time affluence is utilized in health research and to offer theoretical and methodological implications for future studies.

## 2 Method

This study employed a scoping review methodology to systematically map the literature and identify key concepts, research characteristics, and gaps that are related to the study topic (16). This method allows the present study to comprehensively examine how time affluence is conceptualized, operationalized, and empirically examined in relation to health. The scoping review followed the five-stage methodological framework proposed by Arksey and O'Malley (17). The reporting of this review adheres to the Preferred Reporting Items for Systematic Reviews and Meta-Analyses extension for Scoping Reviews (PRISMA-ScR) guidelines (18).

### 2.1 Identifying the research question

This scoping review examines how time affluence is conceptualized and studied in relation to health and wellbeing. The research questions presented above focus on the identification of key conceptual definitions, research designs, and measurement approaches, as well as on producing a summary of reported health-related outcomes. This approach enables a systematic mapping of existing evidence and an identification of research gaps for future studies.

### 2.2 Identifying relevant studies

To identify relevant studies, a comprehensive strategy for a literature search was developed in consultation with an experienced researcher familiar with the study of time use and health. Electronic databases within the fields of psychology, public health, sociology, and economics, including Science Direct, Web of Science, and PubMed, were systematically searched with combinations of keywords related to time affluence and health. The search strategy adopted used Boolean operators (AND, OR) to combine search terms such as “time affluence” and “discretionary time” with health-related terms including “health,” “wellbeing,” “quality of life,” “life satisfaction,” “mental health,” “physical health,” and “health behavior.” The final search was conducted on January 19, 2026.

### 2.3 Study selection

The selection of studies proceeded in multiple stages to ensure that only studies that are relevant to time affluence and health were included. The records that were retrieved from database searches were imported into a reference manager, and duplicate records were removed. In total, 18,700 records were identified through these searches. After 16,460 duplicates were removed, 2,240 unique records remained for screening. The titles and abstracts of these records were examined to assess their relevance to the conceptual and empirical focus on time affluence and its association with health or wellbeing. In this stage, 2,165 records were excluded because they did not meet the inclusion criteria, leaving 75 articles for full-text assessment.

The full texts of the articles were evaluated against the following predefined inclusion criteria: (1) written in English; (2) full-text available; and (3) explicit use of time affluence, perceived time sufficiency, or a closely related construct conceptualizing time as a subjective or experiential resource, along with a conceptual or empirical link to health, wellbeing, or quality of life, including physical health, mental health, health behaviors, or psychosocial wellbeing outcomes.

**Figure 1:**
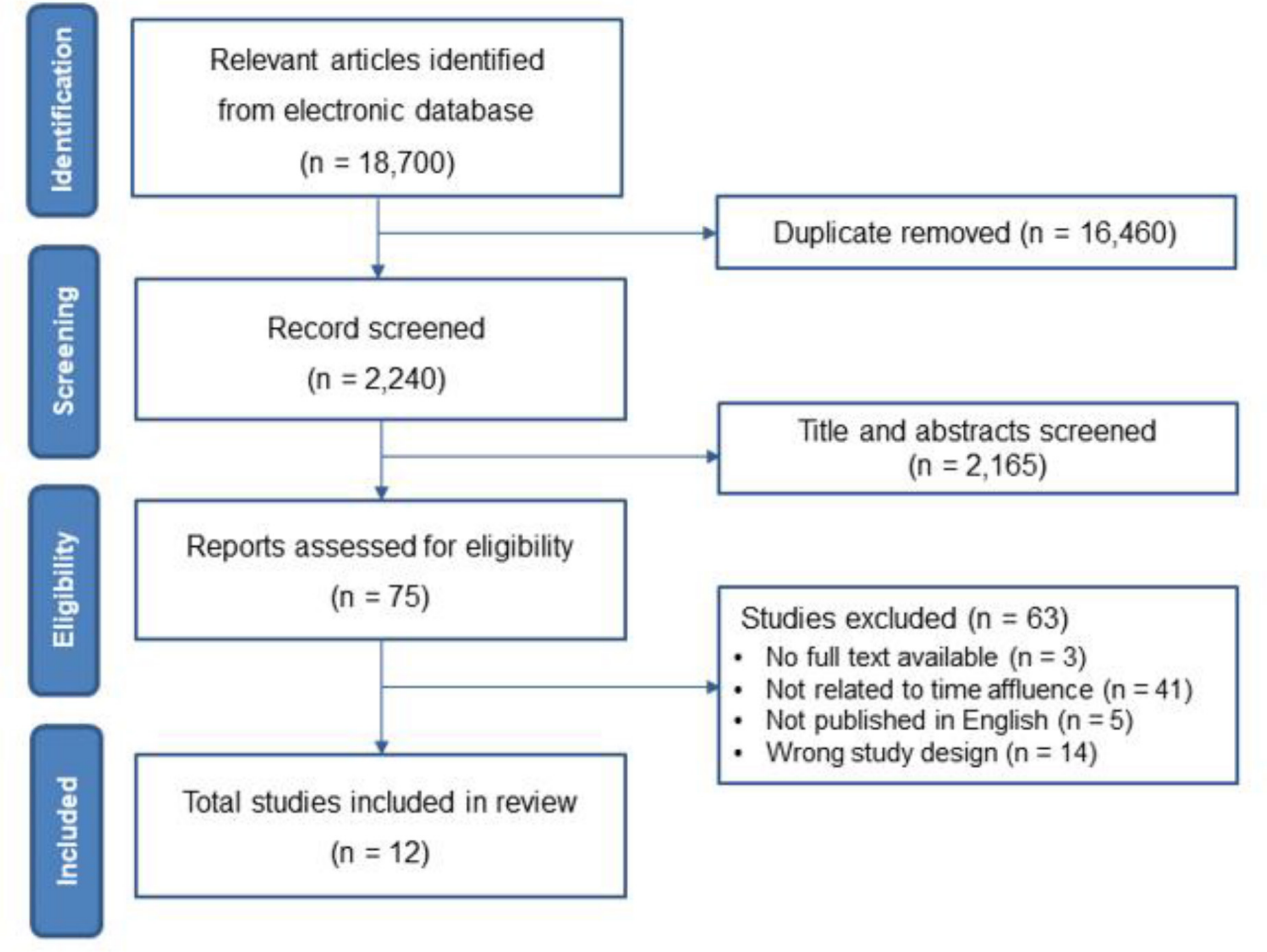
PRISMA flow diagram of the study selection process.

Studies were also excluded if they did not operationalize time affluence (or a clearly analogous construct), were not published in peer-reviewed journals, or did not examine any connection to health or wellbeing. At the full-text stage, 63 studies were excluded using these criteria, resulting in the final inclusion of 12 studies in the review. The complete process of selection process is presented in the PRISMA-ScR flow diagram (Figure 1).

### 2.4 Charting the data

Following the selection of the studies, their data were systematically charted to enable structured comparison and synthesis. A data-charting framework was developed to extract the key information that was relevant to the objectives of the review, with a particular emphasis on how time affluence was conceptualized, defined, and linked to outcomes of health or wellbeing.

The information that was charted included bibliographic details (author, year, and country), study characteristics (research design, sample size, and participant characteristics), definition and theoretical framing of time affluence, measurement or operationalization of time affluence (including whether it was assessed directly or through proxy indicators), the health-related variables examined, principal findings, and the reported limitations. This framework was iteratively refined in the extraction process to ensure conceptual clarity and consistency across studies. Further, studies were categorized as conceptual/theoretical papers, direct empirical investigations, or proxy-based studies, according to how time affluence was defined and measured in them. This structured charting process facilitated a transparent mapping of the literature and supported subsequent synthesis in accordance with the aims of the scoping review.

### 2.5 Collating, summarizing, and reporting results

In the fifth stage, the charted data were collated and synthesized for the provision of a structured mapping of the existing literature on time affluence and its association with health and wellbeing. Consistent with the scoping review approach that was outlined by Arksey and O'Malley (17), the purpose of this stage was not to evaluate the quality of the studies or to generate pooled effect estimates but rather to describe their range, characteristics, and conceptual orientations.

The studies included were organized according to their conceptual and methodological approach to time affluence. In particular, studies were grouped into (1) conceptual or theoretical papers, (2) direct empirical studies that explicitly measured time affluence or perceived time sufficiency, and (3) proxy-based empirical studies that examined related indicators that approximate temporal resources. The characteristics of each group are summarized in Tables 1–3. This classification enabled a systematic comparison of the ways that time affluence has been defined, operationalized, and linked to health-related outcomes across research traditions. In each category, key patterns were identified with respect to theoretical framing, measurement strategies, the types of health and wellbeing outcomes examined, and the reported associations. Particular attention was given to distinguishing those studies that conceptualized time as a subjective psychological resource from those that relied on objective or behavioral indicators as proxies.

**Table 1:** Conceptual and theoretical perspectives on time affluence and health.

References	Disciplinary perspective	Research aim	Research design	Conceptualization of time affluence	Theoretical framework	Proposed pathway to health/wellbeing	Key contribution
Rudd (3)	Social psychology	Conceptually integrates perceived time scarcity and time affluence in wellbeing research	Conceptual narrative synthesis	Subjective psychological perception of having sufficient time, conceptualized in contrast to perceived time scarcity	Time scarcity–affluence continuum; psychological time perception; affective–cognitive mechanisms	Perceived time scarcity increases stress-related cognitive and affective processes, whereas perceived time affluence supports wellbeing through reduced pressure and greater autonomy.	Integrates psychological mechanisms linking perceived time conditions to wellbeing.
Burchardt and Ickler (5)	Political economy	Develops a sociostructural conceptualization of time affluence as a dimension of wellbeing.	Normative conceptual framework	Time as a socially structured resource enabling meaningful, relational, and self-determined activities.	Buen Vivir framework; eudaimonic wellbeing; relational goods; time as a social institution.	Socially available time enables relational participation, autonomy, and quality of life, forming a structural condition for wellbeing.	Positions time affluence as a sociostructural dimension of wellbeing and proposes a time-based inequality framework.
Boniwell and Osin (19)	Positive psychology	Integrates subjective time experience and time use research within a positive psychology framework.	Conceptual narrative review	Subjective perception of having sufficient time contrasts with time poverty.	Subjective time use; time affluence–time poverty continuum; autonomy and self-regulation.	Perceived time sufficiency supports wellbeing through autonomy, meaningful engagement, and reduced stress.	Positions time affluence as a key psychological resource linking subjective time experience and wellbeing.

**Table 2:** Empirical studies that directly measure time affluence and health outcomes.

References	Country	Sample size	Participants	Research design	Health-related variable	Assessment	Result	Limitations
Burke et al. (13)	Türkiye	877	Managers and professionals (manufacturing sector)	Cross-sectional survey	Psychological wellbeing (psychosomatic symptoms, life satisfaction)	MATAS	Positive association between time affluence and psychological wellbeing.	Cross-sectional design
Schaupp and Geiger (23)	Germany	96	Adults in the MBSR intervention and active control (physical exercise) groups	Quasi-experimental longitudinal (pre–post) with active control (non-randomized)	Mindfulness: subjective wellbeing	MATAS	MBSR increased time affluence and subjective wellbeing; time affluence partially mediated wellbeing improvement.	Non-randomized design, self-report measures only.
Kasser and Sheldon (4)	United States	Four independent samples (N = 80–1,178)	Adult employees	Multi-study empirical design (cross-sectional surveys; scale development/validation)	Subjective wellbeing (job, family, and life satisfaction)	MATAS	Time affluence is positively associated with subjective wellbeing independent of income; partial mediation via mindfulness and psychological need satisfaction.	Cross-sectional design; self-report measures; primarily US samples.
LaJeunesse (20)	United States	786	University employees commuting to work (multiple commuting modes)	Cross-sectional survey	Affective wellbeing (stress, commute satisfaction, and calmness); competence	MATAS	Mindfulness is associated with higher time affluence and lower stress; time affluence mediated positive affective appraisals of commuting.	Cross-sectional design; self-report measures; single institutional sample.
Geiger et al. (22)	Germany	1,907	Adults (employed, students, retired, and unemployed)	Cross-sectional survey	Life satisfaction	GTWS (adapted measure)	Mixed associations: sufficient time, unhurried pace, and synchronization are positively related to life satisfaction; plannability and sovereignty are negatively related.	Cross-sectional design; physical health outcomes not assessed; limited subscales; adapted measure
Manolis and Roberts	United States	1,329	Public high school students (adolescents)	Cross-sectional survey (hierarchical regression; moderation analysis)	Subjective wellbeing (life satisfaction, happiness)	MATAS	Materialism and compulsive buying were negatively associated with subjective wellbeing; time affluence moderated (buffered) these negative relationships.	Cross-sectional design; self-report measures; adolescent sample from one geographic region.

**Table 3:** Proxy-based empirical studies on time availability and health outcomes.

References	Country	Participants	Research design	Health-related variable	Assessment	Result	Limitations
Aknin et al. (14)	United States	Multiple countries	Multiple cross-sectional analyses (secondary data)	Life satisfaction (subjective wellbeing)	Discretionary time (hours; proxy indicator of time availability)	Inverted U-shaped association: both low and excessive discretionary time are linked to lower life satisfaction.	Cross-sectional design
Sharif et al. (8)	United States	Secondary datasets (NSCW, ATUS) and adult experimental samples	Multi-study design (two cross-sectional analyses; two experimental studies)	Subjective wellbeing (life satisfaction, happiness)	Discretionary time (hours; proxy indicator of time availability)	Inverted U-shaped association: low and high discretionary time are linked to lower subjective wellbeing.	Cross-sectional design
Tanaka et al. (24)	Japan	Japanese preschool children and mothers	Cross-sectional survey	Childhood obesity (BMI-based classification)	Maternal time availability (structural time constraint proxy; not subjective time affluence)	Lower maternal time availability is associated with higher childhood obesity risk, independent of household economic status.	Cross-sectional design; structural time constraint measure (not subjective perception); parental self-report.

The synthesis was descriptive in nature, focusing on the identification of conceptual patterns, measurement approaches, and gaps in the literature. In line with the purpose of a scoping review, the findings clarify the current state of knowledge and identify areas requiring further theoretical development and empirical research.

## 3 Results

### 3.1 Characteristics of included studies

In all, 12 studies met the inclusion criteria. These were divided into three groups: conceptual or theoretical papers (*n* = 3), direct empirical studies that explicitly measured perceived time affluence (*n* = 6), and proxy-based studies that examined related temporal constructs (*n* = 3). The included studies were conducted primarily in the United States, Germany, Türkiye, and Japan. The empirical investigations were predominantly cross-sectional in design, with limited longitudinal or quasi-experimental approaches. The study samples were largely adult populations, particularly working adults, and fewer studies focused on adolescents or specific groups such as mothers and families.

### 3.2 Conceptualizations of time affluence

Across the included studies, time affluence was most often conceptualized as a subjective perception of having sufficient time and autonomy over its use. Conceptual and theoretical papers identified time affluence as a psychological or relational resource that was embedded within broader wellbeing frameworks. For example, Rudd (3) discussed time affluence with reference to time scarcity, emphasizing its relevance for subjective wellbeing, whereas Burchardt and Ickler (5) situated time affluence within the discussions of temporal inequality and quality of life. Boniwell and Osin (19) similarly considered time affluence to be a component of positive functioning that is linked to perceived control over time.

Empirical studies generally adopted a perceptual definition, operationalizing time affluence as the perception of time sufficiency. Kasser and Sheldon (4) likewise defined time affluence as the subjective sense of having enough time and control over one's own schedule, a conceptualization that is subsequently employed in studies such as Burke et al. (13), LaJeunesse (20), Manolis and Roberts (21), and Geiger et al. (22). These studies centered on individuals' evaluations of whether they had adequate time to engage in meaningful, self-directed, or personally valued activities. Schaupp and Geiger (23), who used the general time wealth scale, similarly conceptualized time wealth as the perceived adequacy of temporal resources.

Overall, proxy-based studies did not consistently explicitly define time affluence but examined related constructs treating time as a resource. For example, Aknin et al. (14) focused on discretionary time and time-saving behaviors, Sharif et al. (8) examined perceived time scarcity, and Tanaka et al. (24) assessed maternal time availability with respect to childcare responsibilities. In these studies, time was primarily conceptualized in structural or behavioral terms, which reflects aspects of temporal resource availability without directly measuring subjective perceptions of time sufficiency.

Overall, the literature demonstrates variation in concepts, particularly in the distinction between time affluence as a subjective psychological experience and time as objectively structured or as a behaviorally operationalized resource.

### 3.3 Operationalization and measurement of time affluence

Measurement approaches varied across conceptual, direct empirical, and proxy-based studies. Conceptual and theoretical papers did not use empirical instruments but incorporated time affluence at a definitional level, describing this as the subjective perception of having sufficient time combined with autonomy over its use (e.g., 3, 5, 19).

Among the direct empirical investigations, perceived time affluence was primarily assessed with the use of self-report scales. Most studies employed the MATAS (4) and measured individuals' perceived time sufficiency and control over the use of their time (4, 13, 20–22). Schaupp and Geiger (23) adopted the General Time Wealth Scale (GTWS), a 12-item instrument that is partially derived from MATAS items but is not identical to it.

Proxy-based studies do not directly assess perceived time affluence but operationalized temporal resources, using indirect indicators. Aknin et al. (14) examined discretionary time and time-saving behaviors, Sharif et al. (8) focused on perceived time scarcity trends, and Tanaka et al. (24) assessed maternal time availability as it is related to caregiving responsibilities. These measures were derived from survey items or large-scale secondary datasets, and they reflected structural or behavioral dimensions of time instead of direct assessments of subjective temporal sufficiency.

In various empirical studies, data collection relied primarily on self-report instruments, where cross-sectional designs represented the dominant methodological approach.

### 3.4 Health and wellbeing domains examined

The health-related outcomes that were examined across the included studies were predominantly psychological in nature. Overall, direct empirical investigations most frequently assessed subjective wellbeing, life satisfaction, stress, and affective states. Kasser and Sheldon (4) examined associations between perceived time affluence and subjective wellbeing, and Burke et al. (13) and Geiger et al. (22) explored the links between time affluence and stress and psychological functioning. Manolis and Roberts (21) analyzed the moderating role of perceived time affluence in adolescents' subjective wellbeing. LaJeunesse (20) likewise examined time affluence in relation to outcomes of work-related wellbeing. Schaupp and Geiger (23) investigated time wealth in relation to workplace stress and psychological outcomes related to work.

Proxy-based studies extended the scope of health outcomes to include psychological and physical indicators. Aknin et al. (14) assessed the relationship between time-related resource allocation and happiness. Sharif et al. (8) examined perceived trends in time scarcity and their association with indicators of wellbeing. Tanaka et al. (24) investigated maternal time availability in relation to childhood obesity, thus incorporating a physical health outcome.

In the investigated studies, mental and psychosocial health outcomes were more frequently examined than physical health ones. Measures of psychological wellbeing, life satisfaction, stress, and affect were predominant, while direct assessments of physical health were comparatively limited.

### 3.5 Observed patterns in associations and study designs

Across the literature reviewed here, associations between time affluence and health-related outcomes were generally reported to show a positive direction. Studies that directly assessed perceived time affluence consistently identified a link between higher levels of perceived temporal sufficiency and greater life satisfaction, lower perceived stress, and more favorable affective states (4, 13, 22, 23). Lower levels of perceived time affluence were associated with lower levels of favorable wellbeing outcomes in several studies (4, 22).

In methodological terms, the empirical research was predominantly based on cross-sectional survey designs. Most studies relied on self-reported data collected at a single time point, and longitudinal or experimental approaches were comparatively limited (4, 13, 20–23). The observed associations therefore reflect patterns identified within predominantly cross-sectional research contexts.

A distinction was observed between studies that conceptualized time affluence as a subjective psychological resource and those that operationalized temporal resources through structurally defined indicators. Direct empirical studies generally relied on validated multi-item self-report instruments to capture perceived time sufficiency and temporal control (4, 22, 23), whereas proxy-based studies drew on structural or behavioral indicators that are derived from large-scale datasets, such as discretionary time, working hours, or time availability in caregiving contexts (8, 14, 24).

Across outcome domains, psychological indicators, including life satisfaction, perceived stress, and affect, were observed more frequently than objective measures of physical health (4, 13, 20, 22, 23). There were few assessments of physical health outcomes within the reviewed research (24).

## 4 Discussion

This scoping review examined the conceptualization, operationalization, and empirically investigation of time affluence in relation to health and wellbeing. Across conceptual and theoretical papers, direct empirical investigations, and proxy-based studies, a consistent pattern was observed: higher levels of time affluence, whether these were defined as perceived temporal sufficiency or were operationalized through related temporal resources, were generally associated with more favorable outcomes in terms of health and wellbeing. Direct empirical studies with validated instruments such as MATAS reported positive associations with life satisfaction and subjective wellbeing, as well as reduced perceived stress (4, 22). Proxy-based investigations likewise indicated that more adequate temporal resources aligned with improved psychological outcomes (8, 14). Likewise, the review identified substantial variation in terms of conceptual and measurement approaches. Conceptual contributions considered time affluence a psychological resource that encompassed perceived sufficiency and autonomy (3, 5), while empirical studies differed in the extent to which they captured subjective experience with reference to structurally defined temporal conditions. While the overall direction of the associations appeared to be consistent, the integration of subjective and structural time dimensions in the literature was uneven.

### 4.1 Conceptual heterogeneity and the need for clarification

The conceptual heterogeneity that was identified in this review is particularly visible in the comparison of the three categories of studies summarized in Tables 1–3. In conceptual and theoretical papers (Table 1), time affluence was consistently framed as a subjective psychological or relational resource, where perceived temporal sufficiency, autonomy, and the capacity to engage in meaningful activities were emphasized. In these frameworks, time affluence was positioned as an experiential construct that was shaped by individual appraisal and broader sociorelational conditions rather than by objective time quantity alone.

However, direct empirical studies (Table 2) primarily operationalized time affluence in terms of self-reported perceptions of time sufficiency and control, which were most frequently measured using the MATAS or a closely related instrument. This pattern suggests an emerging methodological consensus in psychology-oriented research traditions, where time affluence is considered a subjective evaluative construct that was linked to wellbeing outcomes. However, the concentration of studies with the use of similar perceptual measures also indicates a narrowing of conceptual scope, with relatively limited attention to structural or distributive dimensions of time, as emphasized in conceptual work.

A different conceptual orientation was identified in proxy-based studies (Table 3), which relied on structural indicators such as discretionary time, working hours, or caregiving-related time constraints. These studies did not explicitly define time affluence as a subjective experience but treated temporal resources as observable or behaviorally measurable features. Such indicators do allow large-scale population-level analyses, but they generally capture structural availability instead of experiential sufficiency, thereby introducing a conceptual distance from the original psychological definition of time affluence.

Comparison across study types suggests that the field currently draws on at least two partially overlapping conceptual traditions: one that emphasizes subjective temporal experience and another that focuses on structurally distributed temporal resources (15). This coexistence of approaches raises an unresolved question regarding whether subjective time affluence and structurally defined temporal resources form a shared underlying construct or instead distinct dimensions of temporal wellbeing (10). This ambiguity has direct implications for the comparability of measurements and theoretical integration, as findings that are derived from perceptual scales may not be fully commensurate with those that have been obtained from structural proxies (8, 14, 24).

Conceptual clarification may be necessary to support a cumulative development of knowledge. Future research may benefit from an explicit distinction between subjective evaluations of time sufficiency and structurally constrained time conditions while also exploring integrative models accounting for the interaction between these dimensions. This clarification would strengthen theoretical alignment across disciplines and improve the interpretability of evidence that links time affluence to health and wellbeing (6).

### 4.2 Methodological patterns and limitations

The literature was characterized by a predominant reliance on cross-sectional survey designs, a clearly evident pattern across the empirical studies that are summarized in Tables 2, 3. Most empirical studies assessed time affluence and health-related outcomes at a single time point, which limited their ability to establish temporal precedence or to infer causal direction (4, 22). While such designs have consistently documented positive associations, they cannot establish whether perceived time affluence contributes to improved health or whether healthier individuals tend to be more likely to perceive their time as sufficient. In the scope of this review, longitudinal or quasiexperimental designs appeared only rarely [e.g., Schaupp and Geiger (23)], which indicates that temporal dynamics remain underexplored across the evidence base.

There was also limited diversity of measurement approaches. Direct assessments relied primarily on the MATAS or a closely related instrument (4, 13, 22). As shown in Table 2, most direct empirical studies adopted self-report survey designs relying on perceptual measures of time affluence and health-related outcomes, introducing the potential for common method bias. These measures align with conceptualizations of time affluence as a subjective experience, but reliance on self-reported evaluations for both exposure and outcome variables also introduces the potential for common method bias.

Although self-report measures are theoretically appropriate because time affluence is fundamentally conceptualized as a subjective perception of temporal sufficiency and control, the heavy reliance on self-reported assessments may nevertheless introduce methodological concerns. Participants may overestimate or underestimate both their perceived time affluence and health-related outcomes due to social desirability, recall bias, or individual response tendencies. As a result, observed associations may partially reflect shared measurement methods rather than substantive relationships between constructs. Future research would benefit from integrating objective indicators of time use, such as time-use diaries, ecological momentary assessment, or digital tracking data, alongside subjective evaluations.

Furthermore, the relative absence of behavioral indicators, ecological assessments, or mixed methods approaches entails limited opportunity for methodological triangulation within the current literature, which constrains the understanding of how perceived time affluence might correspond to daily temporal practices.

The geographic and demographic concentration of studies further limits their generalizability. Most of the research was conducted in high-income Western countries and focused primarily on working adult populations (4, 22, 23). As indicated in Table 2, few studies included diverse age groups or non-working populations, and cross-cultural comparisons were largely absent. Given the cross-cultural differences in temporal norms, labor structures, and social expectations surrounding time use, it remains uncertain how far the current findings can be extended to other contexts.

Beyond these individual limitations, it is also worth considering the extent to which the methodologies employed were aligned with the underlying research questions. Cross-sectional self-report designs are effective for identifying associations between perceived time affluence and health-related outcomes, yet the theoretical literature conceptualizes time affluence as a dynamic and experiential resource that unfolds over time. Consequently, single-time-point survey designs may capture perceptions of temporal sufficiency while providing limited insight into the processes through which temporal resources influence everyday behaviors and health outcomes. Studies using instruments such as the MATAS offer a standardized means of assessing perceived time affluence within psychological frameworks, but they are less well suited to examining broader structural or contextual dimensions of temporal resources. Proxy-based studies, by contrast, provide broader population-level coverage through large-scale datasets but often sacrifice conceptual precision by relying on indirect indicators of time availability. As a result, each methodological approach appears better suited to addressing certain aspects of time affluence than others. Future research may benefit from greater alignment between conceptual definitions, research questions, and methodological choices, thereby strengthening the overall coherence of the evidence base.

Finally, proxy-based studies entailed additional methodological complexity. Structural indicators, such as discretionary time or working hours, may correlate with perceived time affluence but do not directly capture its experiential dimension (14, 24). The studies that are summarized in Table 3 show that proxy-based approaches often rely on secondary datasets or structural indicators that are designed for broader population analysis instead of for subjective time evaluation. Operationalization differences complicate direct comparison across study type and highlight the need for a greater alignment between structural and perception-based approaches. From a scoping review perspective, it is important that these differences are not interpreted as methodological weaknesses *per se* but rather as reflecting distinct research traditions that are addressing time-related phenomena with different analytical lenses.

### 4.3 Gaps in health domains

In addition to the conceptual and methodological variations, the reviewed literature showed important gaps in the health domains examined. Across study types, research linking time affluence predominantly focuses on psychological indicators, including life satisfaction, perceived stress, affect, and general subjective wellbeing (4, 13, 22). As identified in the empirical studies that are summarized in Table 2, most direct investigations emphasized psychosocial outcomes measured through self-reported indicators of wellbeing. These outcomes are in close alignment with the conceptualization of time affluence as a subjective psychological resource and have shown consistent evidence of positive associations. However, fewer studies have examined physical health indicators or objective measures of health status.

Limited empirical work has explored associations between time-related resources and physical health outcomes, such as weight or health-related behaviors (24). The proxy-based studies summarized in Table 3 suggest that structural time constraints may be relevant for behavioral or physical outcomes, although these approaches rarely assess perceived time affluence directly. As a result, the extent to which perceived time affluence is to be associated with broader public health indicators, including chronic disease risk, health behaviors, or healthcare utilization, has been insufficiently examined. In that temporal constraints and perceived time sufficiency could influence lifestyle practices such as physical activity, sleep, and dietary patterns, further investigation across behavioral and physiological domains should clarify the public health relevance of time affluence.

Moreover, the evidence base assessed here largely emphasizes individual-level psychological outcomes, where less attention is paid to population-level health inequalities or the structural determinants of health. Across the reviewed studies, few analyses explicitly examined how temporal resources can be socially distributed across population groups, in spite of evidence suggesting that time availability could be shaped by socioeconomic status, employment conditions, caregiving responsibilities, and social roles (2, 6). Time affluence can intersect with these factors in ways that shape differential health outcomes. However, these intersections have only been explored indirectly. Greater attention to how time affluence operates in broader social and structural contexts could enhance the understanding of its relevance for health equity.

The concentration of research in psychosocial domains suggests that the health implications of time affluence are only partially mapped. In the context of a scoping review, this pattern reflects the current developmental stage of the evidence base rather than a definitive boundary for the concept itself. Expanding inquiry beyond subjective wellbeing to include behavioral, physiological, and population health indicators could contribute to a more comprehensive understanding of the role that temporal resources play in public health.

### 4.4 Cross-cutting synthesis: convergence, divergence, and theoretical implications

A consistent pattern emerged across all three categories of studies included in this review. Regardless of whether time affluence was conceptualized as perceived temporal sufficiency, structurally available time, or a resource enabling meaningful and relational engagement, greater temporal resources were generally associated with more favorable health and wellbeing outcomes. This convergence is noteworthy because it suggests that temporal resources may represent an important, albeit underexplored, determinant of health across diverse research traditions. At the same time, consistency in findings should not be interpreted as evidence of conceptual agreement. The observed convergence may partly reflect common methodological characteristics among empirical studies, including the predominance of cross-sectional designs, self-report assessments, and psychologically oriented outcome measures, rather than a fully integrated body of evidence. Moreover, no empirical study identified in this review directly compared subjective and objective indicators of time affluence within the same sample. Consequently, the extent to which these approaches capture a common underlying construct remains an unresolved empirical question.

Closer examination reveals that the three research traditions differ not only in measurement but also in their underlying assumptions regarding the nature of time affluence itself. Conceptual and theoretical papers (Table 1) primarily portray time affluence as an experiential and relational resource associated with autonomy, meaningful engagement, and broader concerns of temporal justice (3, 5). Direct empirical studies (Table 2) typically operationalize time affluence as an individual-level psychological appraisal, most commonly measured through instruments such as the Multi-dimensional Time Affluence Scale (MATAS) (4). In contrast, proxy-based studies (Table 3) conceptualize temporal resources as structurally distributed conditions reflected in indicators such as discretionary time, working hours, or caregiving-related constraints (8, 14, 24). These approaches therefore differ in their unit of analysis, their assumptions regarding the mechanisms linking time to health, and the outcomes they prioritize, ranging from subjective wellbeing to population-level health inequalities. Treating findings from these traditions as directly interchangeable may therefore obscure important conceptual distinctions and limit theoretical clarity.

Nevertheless, these differences should not be viewed solely as limitations. Rather, they point toward opportunities for theoretical and methodological advancement. Conceptual frameworks emphasizing temporal autonomy, meaningful engagement, and relational participation (5, 15) provide a foundation for understanding why time affluence may matter for health beyond simple perceptions of having enough time. Direct empirical studies contribute methodological rigor through validated instruments that allow systematic hypothesis testing, while proxy-based studies offer insight into the social distribution of temporal resources and their relevance for health equity (2, 6). Future research would benefit from integrating these perspectives through research designs that combine subjective assessments with objective indicators such as time-use diaries, ecological momentary assessment, or behavioral measures. Such approaches would enable simultaneous examination of the experiential, behavioral, and structural dimensions of time affluence.

The current literature suggests that empirical evidence regarding the health benefits of temporal resources has accumulated more rapidly than theoretical consensus regarding what time affluence actually represents. While researchers increasingly agree that temporal resources are relevant for health and wellbeing, there remains limited agreement regarding the construct itself, its boundaries, and its most appropriate operationalization. Until these conceptual and methodological divisions are more explicitly reconciled, conclusions regarding the health effects of time affluence should be interpreted with caution, as studies may be using the same terminology to describe substantially different temporal phenomena. At the same time, this productive tension provides an important foundation for future theory development and may ultimately contribute to a more coherent and comprehensive understanding of temporal resources as determinants of health.

### 4.5 Limitations and implications for future research

The conceptual, methodological, and substantive gaps that are identified in this review indicate several priorities for advancing research on time affluence and health. In addition to its contributions, this study has several limitations that should be acknowledged. As a scoping review, it mapped the breadth of existing literature rather than formally assessing methodological quality or synthesizing effect sizes (17, 18). Thus, the findings should be considered to be descriptive of current research patterns rather than being definitive evidence of causal relationships.

First, greater conceptual precision could enhance theoretical coherence across disciplines. Clarification of whether time affluence is primarily a subjective psychological resource, a structurally distributed temporal condition, or a construct that integrates both dimensions may improve comparability across studies, supporting cumulative knowledge development (5, 10, 12). Explicit differentiation between experiential and structural dimensions of temporal resources could also reduce conceptual ambiguity in future empirical investigations.

11gSecond, methodological diversification strengthens the evidence base. Longitudinal designs are required to examine temporal ordering and explore whether perceived time affluence precedes improvements in health or whether healthier individuals are more likely to consider their time as sufficient. Experimental and intervention-based approaches could clarify whether modifying the perceptions of time sufficiency influences health behaviors or psychological outcomes. The incorporation of mixed methods, ecological momentary assessments, or objective indicators of time use could enhance understanding of how perceived time affluence corresponds to temporal practices (2).

Third, the expansion of the scope of health outcomes examined could increase the relevance of time affluence research in public health. Future studies could benefit from assessing associations with health behaviors, chronic disease risk, physiological indicators, and healthcare utilization, in addition to psychological wellbeing. This expansion would help determine whether time affluence is a meaningful factor in population health beyond its documented links to subjective outcomes (6, 7).

Finally, greater attention is warranted to distributional patterns. The investigation of how time affluence varies across socioeconomic groups, employment conditions, caregiving roles, and cultural contexts could clarify its role in health inequalities (2, 5). The understanding of whether time affluence mediates or moderates social gradients in health could contribute to broader discussions of temporal resources as potential social determinants of health.

## 5 Conclusion

This scoping review systematically examined the ways that time affluence has been conceptualized, measured, and linked to health and wellbeing in a range of research traditions. Across conceptual, direct empirical, and proxy-based studies, a pattern emerged in which higher levels of perceived temporal sufficiency were associated with more favorable outcomes for psychological health. However, substantial variation was observed in how the definition and operationalization of time affluence, reflecting an ongoing conceptual ambiguity in the literature. The evidence base was further characterized by the predominance of cross-sectional designs, reliance on self-report measures, and a primary focus on psychosocial outcomes. Indicators of physical health, behavioral pathways, and population-level inequalities received less attention. These patterns suggest that, while time affluence is relevant for subjective wellbeing, its broader public health implications have been only partially explored. The findings underscore the importance of greater conceptual precision, methodological diversification, and expanded outcome domains for future research. Clarifying the role of temporal resources in health frameworks could contribute to a more comprehensive understanding of how time-related conditions shape individual and population health.
